# Supplementary material for: Study of Cytotoxicity and Internalization of Redox-Responsive Iron Oxide Nanoparticles on PC-3 and 4T1 Cancer Cell Lines
Source: Pharmaceutics. 2022 Dec 30;15(1):127. doi: 10.3390/pharmaceutics15010127 (PMC9864410; doi:10.3390/pharmaceutics15010127)
Supplement: Supplementary file 1 [file pharmaceutics-15-00127-s001.zip › pharmaceutics-2101657-supplementary.pdf]

Study of Cytotoxicity and Internalization of Redox-Responsive Iron Oxide Nanoparticles on PC-3 and 4T1 Cancer Cell Lines

Timur R. Nizamov [1,*,†], Artem R. Iliasov [1,†], Stepan S. Vodopyanov [1,2], Irina V. Kozhina [1], Igor G. Bordyuzhin [1], Dmitry G. Zhukov [1], Anna V. Ivanova [1], Elizaveta S. Permyakova [1], Pavel S. Mogilnikov [1], Daniil A. Vishnevskiy [3], Igor V. Shchetinin [1], Maxim A. Abakumov [1,3] and Alexander G. Savchenko [1]

[1] Department of Physical Materials Science, National University of Science and Technology (MISIS), Moscow 119049, Russia
[2] Department of Invertebrate Zoology, Faculty of Biology, Lomonosov Moscow State University, Moscow 119234, Russia
[3] Department of Medical Nanobiotechnology, N.I. Pirogov Russian National Research Medical University, Moscow 117997, Russia
* Correspondence: nizamov.timur@gmail.com
† These authors contributed equally to this work.

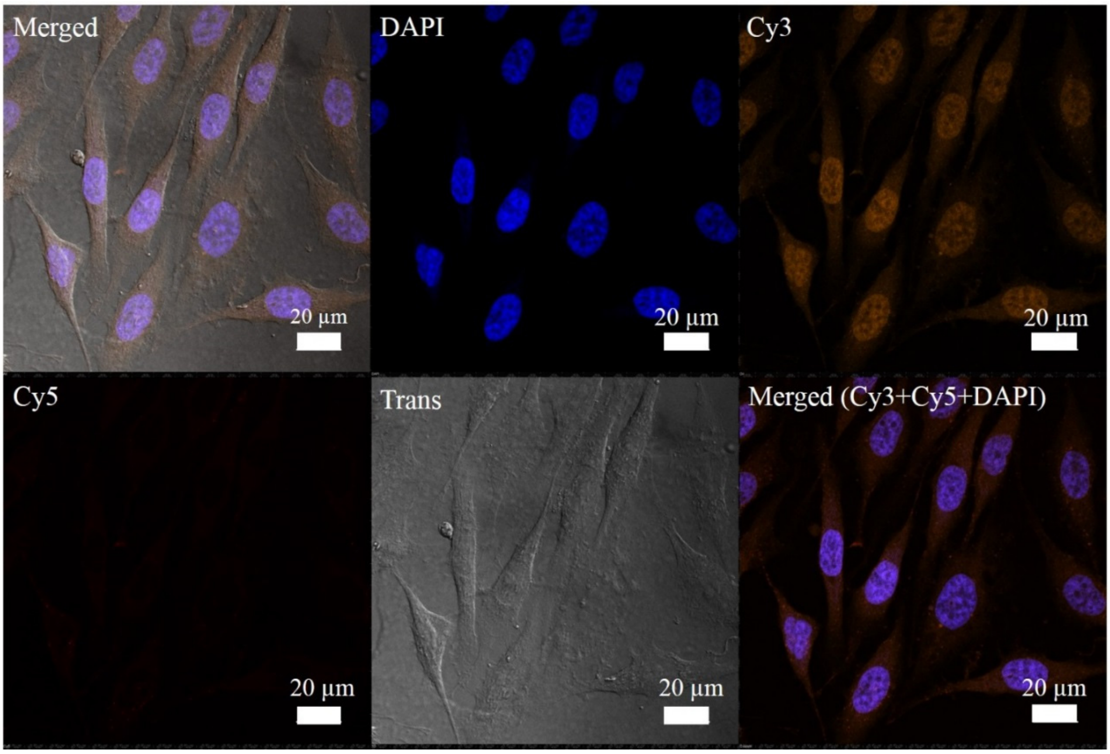

Figure S1. PC3 cells incubated with Cy3 and Cy5 fluorescently labeled MNP-SS nanoparticles for 1 h.

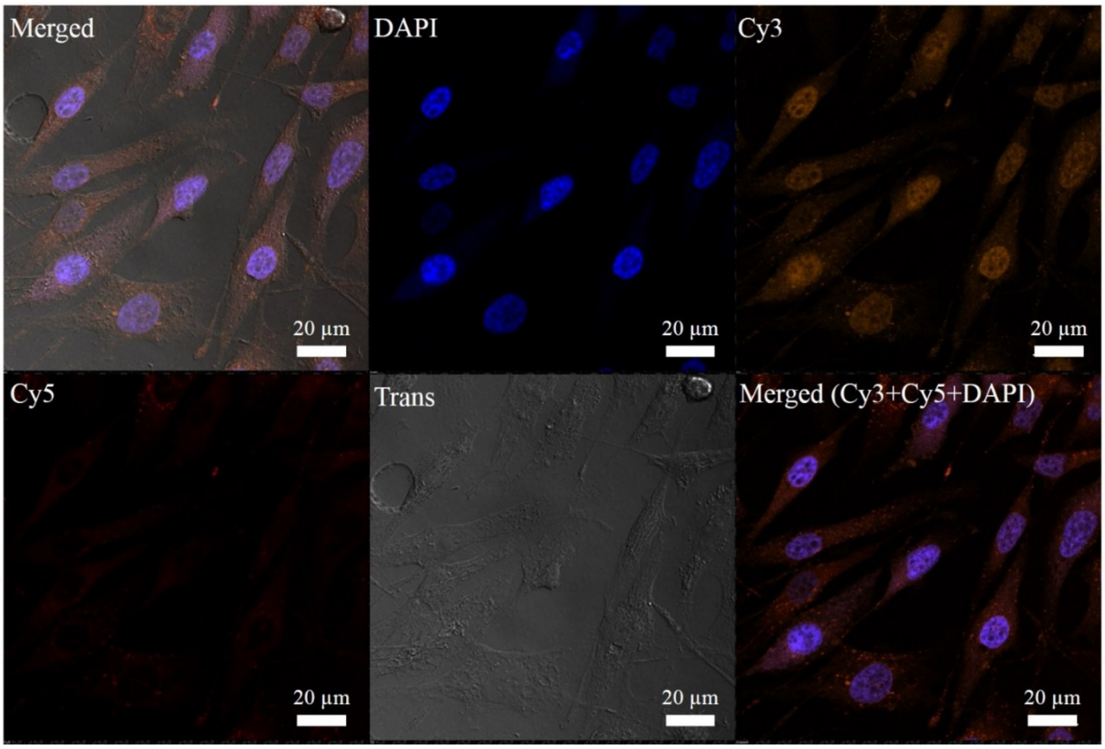

Figure S2. PC3 cells incubated with Cy3 and Cy5 fluorescently labeled MNP-SS nanoparticles for 4 h.

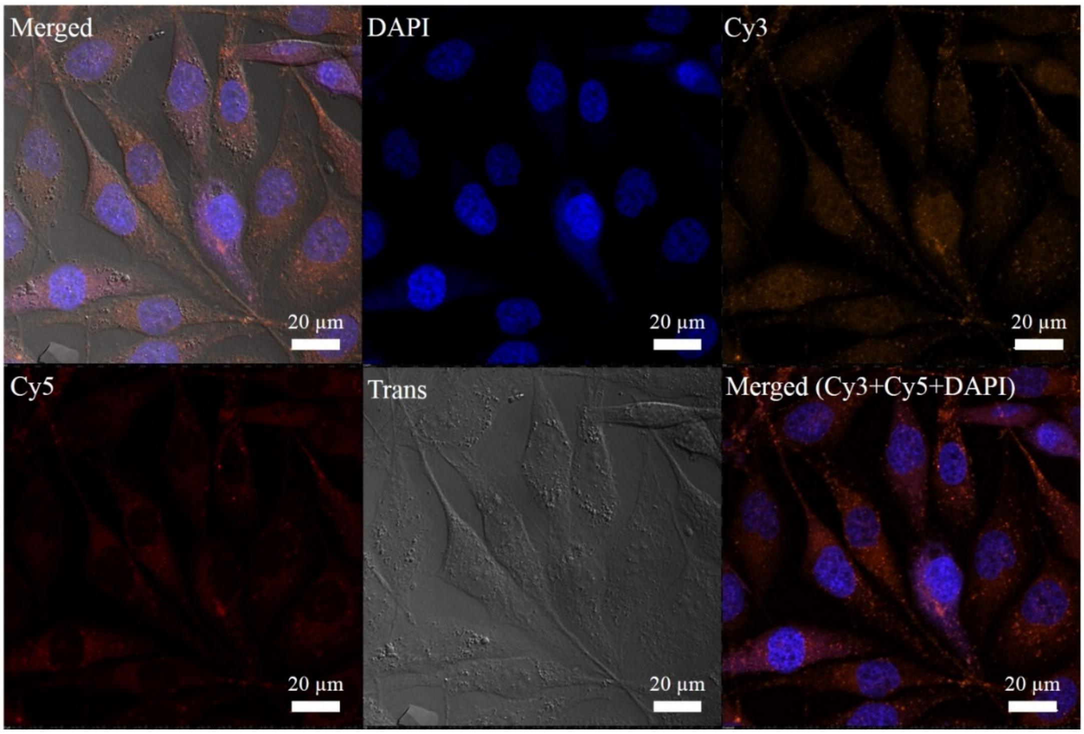

Figure S3. PC3 cells incubated with Cy3 and Cy5 fluorescently labeled MNP-SS nanoparticles for 24 h.

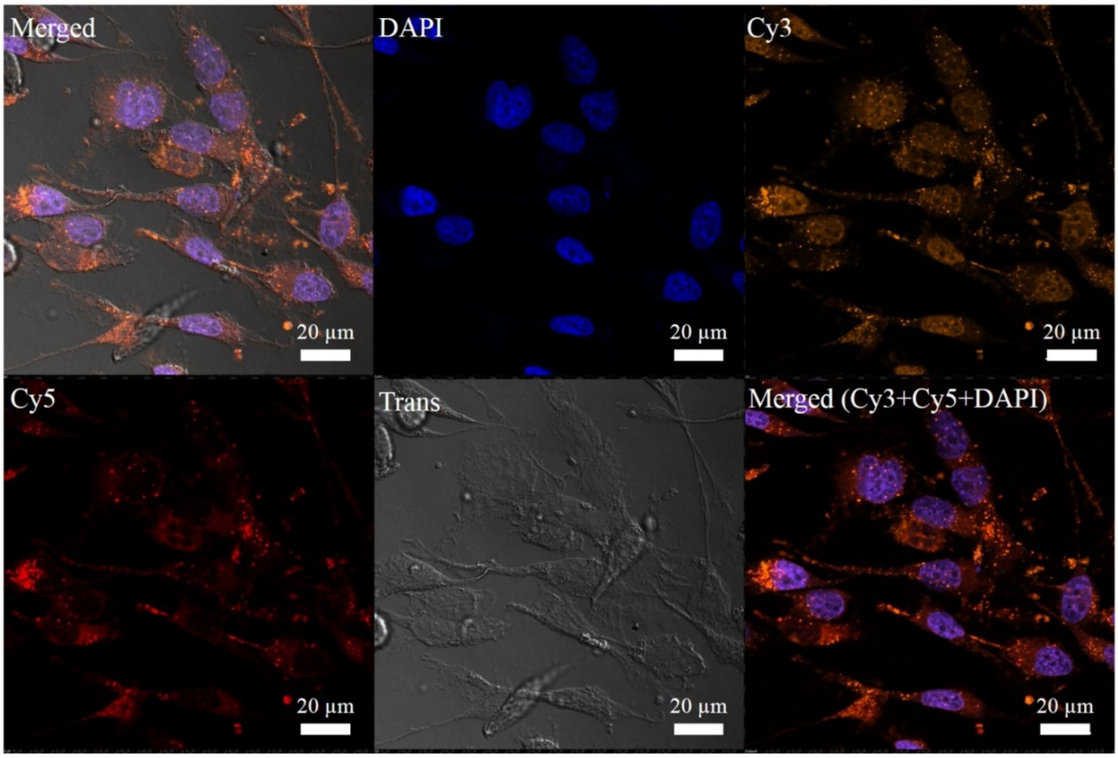

Figure S4. PC3 cells incubated with Cy3 and Cy5 fluorescently labeled MNP-SS nanoparticles for 48 h.

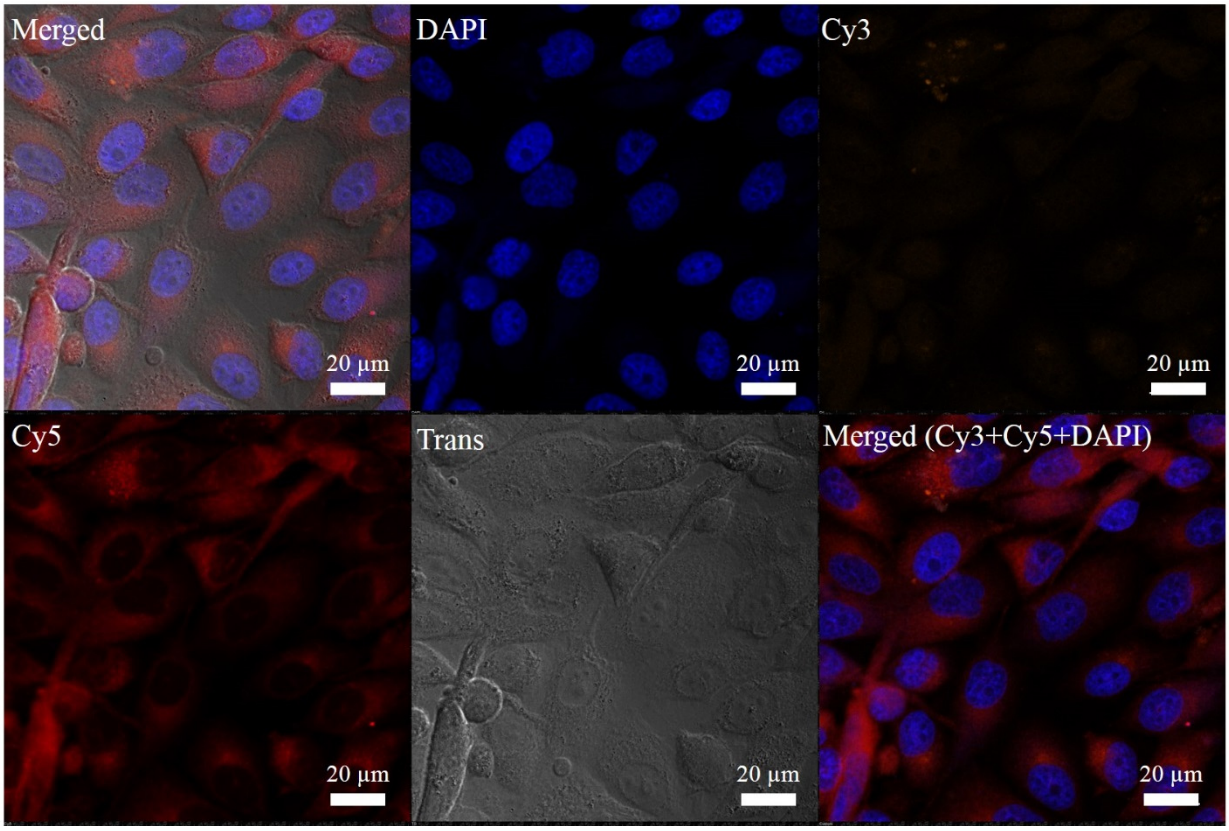

Figure S5. PC3 cells incubated with Cy3 and Cy5 fluorescently labeled MNP-SS nanoparticles for 72 h.

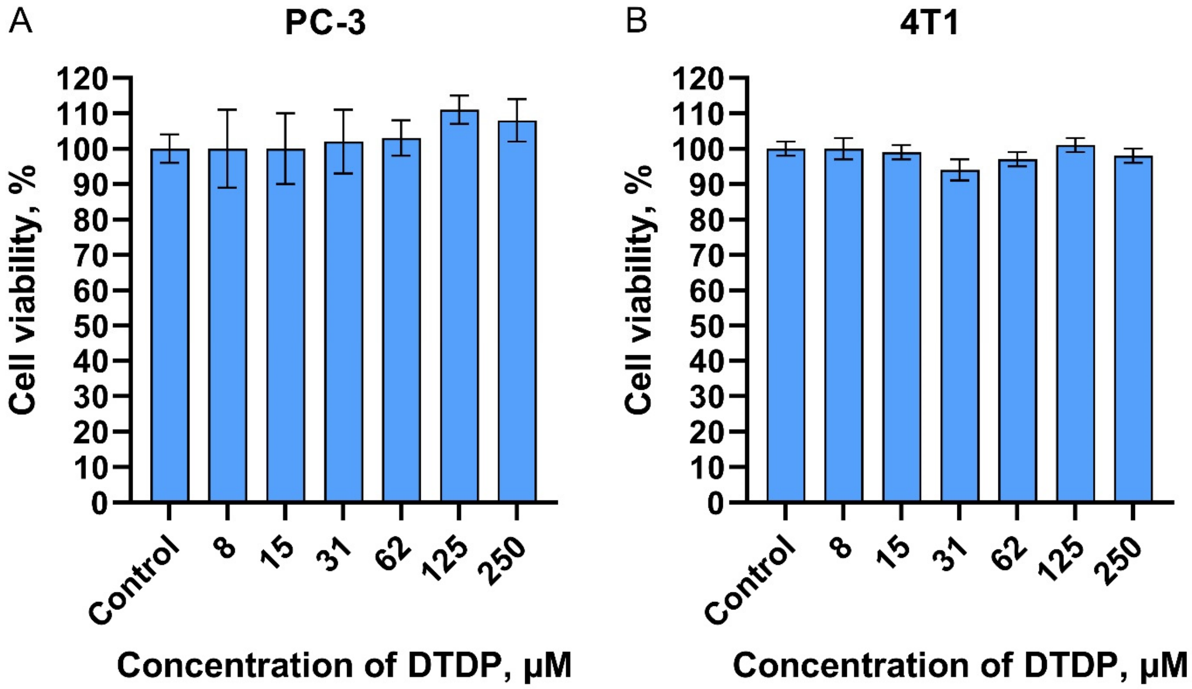

Figure S6. Evaluation of cytotoxicity of pristine DTDP on PC-3 (**A**) and 4T1 (**B**) cells.

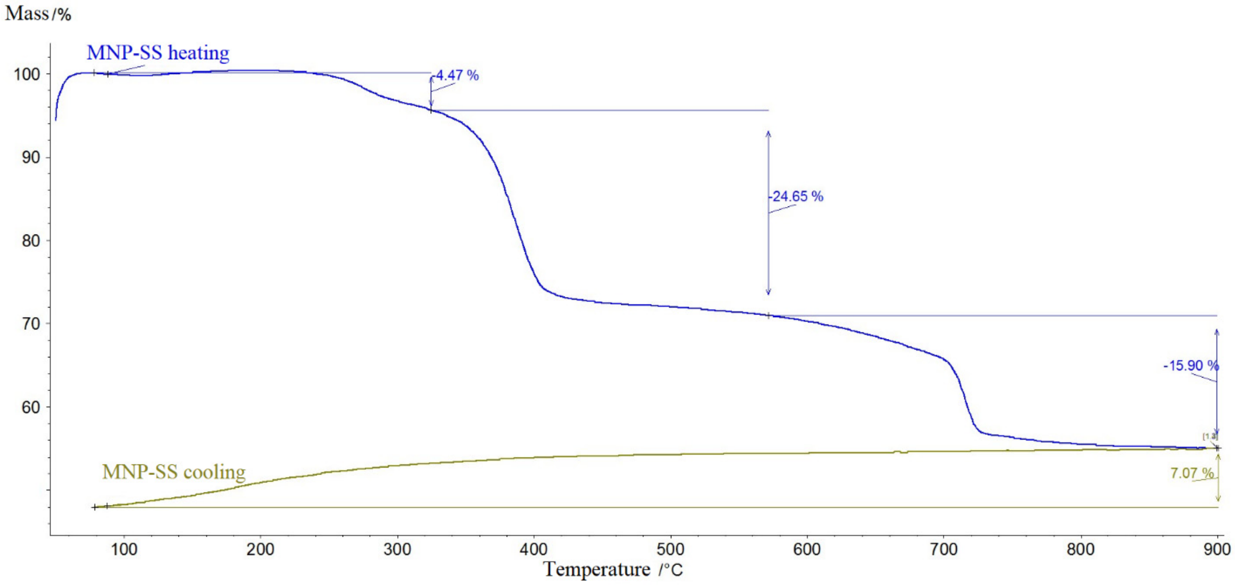

Figure S7. TGA data for the MNP-SS sample.
